# When the lights went out: impacts of the April 2025 Iberian blackout on the Portuguese National Health Service sovereignty - a reflection on national defence, health sovereignty, risk, and infrastructural dependency

**DOI:** 10.3389/fpubh.2025.1630933

**Published:** 2025-07-09

**Authors:** Francisco Goiana-da-Silva, Daniel Madureira-Fonseca, Duarte Tude Graça, Maria Moitinho De Almeida, Miguel Cabral Pinho, Juliana Sá, Rui Moreira, Luís Cabral, Nelson Pereira, Alexandre Morais Nunes, Alexandre Lourenço, Jaime Branco, Hutan Ashrafian, Fernando Araújo, Ara Darzi

**Affiliations:** ^1^Centre for Health Policy, Institute of Global Health Innovation, Imperial College London, London, United Kingdom; ^2^NOVA Medical School, Universidade NOVA de Lisboa, Lisbon, Portugal; ^3^Faculdade de Ciências da Saúde, Universidade da Beira Interior, Covilhã, Portugal; ^4^NATO, Supreme Headquarters Allied Powers Europe, Mons, Belgium; ^5^Faculdade de Medicina, Universidade de Lisboa, Lisbon, Portugal; ^6^Centre for Research on the Epidemiology of Disasters, Institute of Health and Society, UCLouvain, Ottignies-Louvain-la-Neuve, Belgium; ^7^ULS São João, Porto, Portugal; ^8^Department of Internal Medicine, ULS Santo António, Porto, Portugal; ^9^Department of Medical Sciences, University of Aveiro, Aveiro, Portugal; ^10^Portucalense Business School, Universidade Portucalense, Porto, Portugal; ^11^Servico Regional de Protecao Civil e Bombeiros dos Acores, Ilha Terceira, Portugal; ^12^Portuguese College of Emergency Medicine of the Portuguese Medical Association, Lisbon, Portugal; ^13^Centre for Public Administration and Public Policies, Institute of Social and Political Sciences, University of Lisbon, Lisbon, Portugal; ^14^National School of Public Health (NOVA NSPH), Universidade NOVA de Lisboa, Lisbon, Portugal; ^15^ULS Coimbra, Coimbra, Portugal; ^16^Faculty of Medicine, Department of Surgery and Cancer, Imperial College London, London, United Kingdom; ^17^Faculty of Medicine, University of Porto, Porto, Portugal

**Keywords:** health system resilience, emergency preparedness, infrastructure, energy blackout, extreme events

## Abstract

The April 2025 blackout in the Iberian Peninsula severely disrupted The Portuguese National Health Service (NHS). It led to failures for over eight hours in power supply, digital systems, telecommunications, and inter-institutional coordination. Hospitals operated on limited generator capacity, essential medical equipment was triaged, and digital health records became inaccessible. Cold storage failures endangered temperature-sensitive medicines, while emergency communications and transport systems were severely compromised. This perspective article based on first-hand experience and grey literature proposes a first rapid analyses on the blackout as through the World Health Organization’s Health Emergency and Disaster Risk Management Framework. Advocating that health care resilience must then be acknowledged as a fundamental area of national security the article calls for regulatory improvement, decentralized energy solutions, digital redundancy, and integrated command structures linking health, civil protection, and defence sectors. It offers further insights into building anticipatory health systems capable of withstanding future complex disruptions.

## 1 Introduction

On April 28th, 2025, an unprecedented widespread power outage swept across the Iberian Peninsula, triggering one of the most significant infrastructural failures in recent memory and leaving tens of millions of people without power for over 8 h (1–3).

In Portugal, beyond paralysing essential infrastructures, the blackout had immediate and far-reaching consequences for the Portuguese National Health Service (NHS). As hospitals, health centres, and critical digital care platforms lost power, the phenomenon exposed not only technical vulnerabilities but also profound questions on national preparedness, infrastructural autonomy, and health sovereignty. For health professionals, this was not simply a power failure, it was a system stress-test that pushed the boundaries upon which modern healthcare relies on a daily basis (1, 4, 5).

In the context of growing geopolitical instability, where the spectre of conflict looms and possible threats to national security are multiplying, healthcare systems must be recognized not simply as service providers but as pillars of national defence and sovereignty (6). The April 2025 blackout exposed the susceptibility of the health sector to a range of potential threats from cyberattacks and energy disruption to the physical sabotage of critical infrastructures. While official statements have ruled out the possibility of deliberate aggression, the incident functioned as an unplanned stress test, revealing the structural and operational fragilities of Portugal’s health system and underscoring the urgent need to fortify health infrastructures and preparedness (3, 4, 7).

Experiences from other disaster contexts show that hospitals often continue to operate despite major disruptions, but resilience depends not just on physical infrastructure, but also on institutional flexibility, communication systems, and staff preparedness. For instance, the 2015 Nepal earthquake revealed that even structurally intact hospitals were hampered by logistical, administrative, and human resource challenges, exposing deep systemic vulnerabilities under stress (8). Furthermore, as Wasiullah et al. (9) demonstrate, resilience in crisis is not merely a matter of recovery capacity but of pre-existing institutional robustness and governance structures that can absorb and adapt to unexpected shocks. This blackout also took place at a particular relevant moment, since about one month before, the European Commission launched its Preparedness Union Strategy to support Member States and enhance Europe’s capability to prevent and respond to emerging threats (10). Thus, furthering previous global references such as the United Nations’ Sendai Framework for Disaster Risk Reduction 2015–2030 (11), and, in the specific field of health, the World Health Organization (WHO)‘s Health Emergency and Disaster Risk Management (Health EDRM) Framework (12).

Given the unprecedented and recent nature of this event, this article relies considerably on: (i) the first-hand knowledge and experience from the authors on the events that took place, in several situation rooms, from their executive and leadership positions in relevant institutions impacted by the described events; (ii) grey literature from trustworthy news outlets. For this, a scoping review of grey literature was done until a saturation point was achieved for relevant new topics in additional sources. The lack of peer-reviewed articles on this topic exposes a pre-existing gap in the literature, underlining the importance of knowledge sharing with the scientific community, such as this perspective article. Given the involvement of several of the co-authors in the coordination of the response to this extreme event, this manuscript provides not only an external critical analysis of what happened but also a public accountability exercise with and acknowledgement of the systems’ limitations. This perspective article examines the April 2025 blackout through the lens of health system resilience and sovereignty. It argues that energy security, infrastructure governance, and digital autonomy must be reframed as core health policy issues. By drawing on Portugal’s experience and positioning it within global debates on digital fragility and systemic risk, this perspective seeks to contribute to a broader reimagining of what it means to build a sustainable and sovereign National Health Service in the 21st century.

## 2 Vulnerabilities exposed during the April 2025 Iberian power outage

To further explore the impacts of the blackout in the healthcare provision Table 1 shows a timeline of major events during the blackout.

**Table 1:** Timeline of major events during the blackout.

Time (BST/UTC + 1)	Key event
11:33	A nationwide power blackout occurs in Portugal, later confirmed as part of an Iberian-wide electrical grid failure.
11:47	The National Authority for Emergency and Civil Protection initiates contact with Portugal’s electricity transmission system operator and the national low-voltage distribution company to assess the situation.
11:48	Emergency communication systems are engaged; national emergency medical services and municipal firefighting forces are alerted.
11:50	The transmission system operator reports a major outage of unknown origin affecting the entire national territory.
12:04	Authorities are informed that the blackout extends across the Iberian Peninsula, with a potential resolution timeframe of up to 24 h.
12:05	A strategic crisis communication plan is activated; mobile network operators are contacted to evaluate the extent of telecommunications impact.
12:18	Authorities determine the need to create a dedicated National Operational Structure for Internal Security, once the event’s classification is clarified (i.e., civil protection vs. internal security incident).
12:27	A formal request for operational intelligence is sent to the European Civil Protection Mechanism. The response provides only generic information about the availability of emergency generators.
12:30	The Civil Protection authority decides to activate the National Operational Coordination Centre (CCON), with operations to begin at 13:30. Tactical command facilities are placed on backup power as a redundancy measure.
12:47	Detailed situational updates and operational instructions are disseminated to the full network of national civil protection units.
13:05	The first public communication is released to the national media.
13:10	The Secretary of State for Civil Protection arrives at the command coordination facility.
13:15	The blackout is now confirmed to be generalized nationwide. Priority is given to securing fuel supplies for hospitals, dialysis centres, and patients dependent on respiratory support. Contingency plans are recommended for hospitals, nursing homes, and long-term care units. Status updates are also requested from airports, underground transport systems, data centres, and fuel supply chains.
13:30	The national operational coordination centre becomes fully active. Around 30 public and private entities are represented, including the Armed Forces, national police forces, emergency medical services, intelligence and transport authorities, water utilities, fuel distributors, and major telecommunications providers.
13:45	An urgent needs assessment for generators and fuel is completed, prioritising healthcare facilities. Traffic gridlocks caused by inoperative traffic lights necessitate police escorts for fuel delivery vehicles.
14:30	Activation of emergency response plans in 23 municipalities and one district (Coimbra).
16:30	A comprehensive list of critical facilities in need of generator support and fuel resupply is finalized. Major hospitals report dangerously low fuel levels for their backup systems.
17:15	Preparation begins for a nationwide SMS alert to the population, despite limited information still being available.
18:00	The first SMS alerts are sent. Only around 2.5 million individuals receive the message, representing less than 50% of the expected reach due to system overload.
18:28	A public advisory is posted on social media, urging people to avoid unnecessary travel.
19:30	A technical-operational bulletin is issued at “orange” alert level. Although some municipalities (e.g., Abrantes, Santarém) show signs of partial power recovery, widespread disruptions continue. Contingency planning expands to include water supply and support for critical infrastructure.
20:30	The Council of Ministers issues a resolution declaring a national energy crisis and activates the Strategic Fuel Supply Network.
23:15	The Minister of Internal Administration arrives at the coordination centre and participates in a situation briefing that continues until approximately 01:30.
00:30–06:00	Power supply is gradually restored across the country during the night. The 23 municipal emergency plans are progressively deactivated. The national coordination structure remains in operation for approximately 24 h.

Source: Civil Protection National Authority according to a major news outlet (35).

The power outage exposed a set of issues that require greater attention from the government, the different institutions, professionals and citizens. These are particularly important when we consider the high risk associated with a seismic event, where the full operation of healthcare services is crucial due to the likely need to assist affected citizens. Among other factors, the following points highlight those that proved to be the most sensitive during this “test,” while still trying to use the WHO’s Health EDRM Framework (12).

### 2.1 Policies, strategies and legislation

While Spain promptly declared a state of emergency to streamline its national response and ensure coordinated resource management, Portugal refrained from activating similar mechanisms, exposing a lack of harmonization between the two countries’ crisis response frameworks (13).

Portugal is yet to adopt resilience directives that assert service continuity during crises, following Nordic models such as Norway’s legally binding backup power obligations and Finland’s contingency planning frameworks. Portugal lacks binding national regulations that enforce minimum backup power requirements for telecommunications infrastructure during crises, placing it out of alignment with Article 95 of the EU Electronic Communications Code, which permits Member States to mandate network resilience for emergency services. In contrast, Nordic countries such as Norway and Finland enforce regulatory standards requiring 4 to 24 h of backup power at critical mobile sites, routine stress testing, and contingency planning obligations supervised by national telecom authorities (14, 15). Current Portuguese regulation remains misaligned with Article 95 of the EU Electronic Communications Code, which allows Member States to enforce resilience for critical services.

### 2.2 Planning and coordination

Traffic light failures, road congestion, and communication breakdowns with ambulance dispatch centres probably caused delayed emergency transport and patient transfers. as the literature already points to in situations where there are specific changes to normal traffic (16). Reports indicated that cities were gridlocked during the blackout, which may have had implications for fuel transport and emergency services (2). A major cause was the failure of SIRESP, the national emergency communication system, which left services unable to coordinate effectively during the blackout. In response, the government announced the urgent creation of a team to replace the system, admitting its failure under pressure (17).

The current and temporary Portuguese NHS Executive Board headquarters had no power generator, rendering both landlines and IT infrastructure inoperative. The Portuguese NHS CEO was therefore forced to relocate to the National Institute of Medical Emergency (INEM) to restore secure communication.

Hospitals activated their contingency plans, prioritizing core services such as emergency departments, intensive care, and life-support systems. While some variation is expected, the lack of coordination across hospitals within the NHS network created avoidable disruptions, and it is unclear how well the centralization of this information was achieved (18). Decisions made in isolation, such as suspending diagnostics or redirecting patients, had a direct impact on neighbouring units, since patients move between hospitals and rely on the system as a whole. This fragmentation exposed the need for greater coordination and shared protocols. Although hospital-level autonomy in emergencies is necessary, contingency plans must be well-designed, regularly tested, and aligned across institutions to prevent operational conflicts. The blackout offered a real-life drill that should now inform concrete improvements.

For example, due to asymmetries in the prioritization of energy allocation for high-demand diagnostic equipment, established medical referral networks for patients in critical condition - such as those suffering from stroke and other acute cardiovascular events - were disrupted and required *ad hoc* reconfiguration. In central Portugal, the Unidade Local de Saúde de Coimbra was compelled to receive stroke patients from neighbouring local health units that had unilaterally suspended the operation of essential diagnostic technologies, including CT and MRI scanners, in response to localized energy constraints.

### 2.3 Human resources

Some digital tools used for staff scheduling and shift coordination became inoperative. Telecommunication companies’ service was irregular or inoperable, putting the availability of health professionals in question. In the case of the professionals that were arriving, they were doing so without clear instructions, causing overstaffing in some areas and shortages in others. Manual reallocation was necessary, as well as face to face communication was often necessary placing additional pressure on local leadership and administrative teams. While this was still a possibility in Hospitals due to the centralization of teams in the same space, the case was very different for primary healthcare facilities, where some teams were unreachable by telecommunication and physical dislocation was not recommended due to the traffic gridlocks.

### 2.4 Financial resources

As mentioned below, there was a quite diverse reality across the country in what regards the conditions of the different health institutions. In some the ability to respond to this crisis was very limited while in others it was possible to keep the normal operations. This highlights the need for greater investment in several health institutions. It also prompts the concern about the current ongoing health facilities constructions in progress, since a great deal of investment is being done but there is no certainty about the role these new facilities can play in future crisis.

### 2.5 Information and knowledge management

The shutdown of mobile networks, landlines, and internet services resulted in severe constrains of institutional communication. Hospitals were unable to coordinate with each other, civil protection authorities, or emergency services. In some facilities, even internal paging and staff communication systems were inoperative. Critically, the national emergency number (112) became temporarily inaccessible, leaving the public without a functioning lifeline during a crucial period, although the Minister of Health assured that the calls that were not immediately attended to were later on addressed, leaving no one without a reply (19). This communications blackout had cascading consequences for patient safety and operational continuity (19).

The blackout exposed the critical vulnerability of Portugal’s clinical IT infrastructure. Electronic Health Records (EHRs), digital prescribing systems, and diagnostic imaging platforms became inaccessible within minutes, severely disrupting clinical workflows. Although backup generators preserved limited functionality in some institutions, many were forced to revert to paper-based procedures (4). Although this is and should be a routine procedure in contingency situations, the lack of adequate preparedness, might lead to situations compromising the continuity and safety of care, particularly for patients with complex or chronic conditions. This regression might not only increase the risk of clinical error, incomplete documentation, and duplicated treatments, but also echoed broader concerns raised in comparable systems. As observed in the NHS, failing IT infrastructure has been shown to undermine safe healthcare delivery, particularly when digital systems lack the resilience required to function under duress (20).

### 2.6 Risk communication

The communication with the population suffered also in great measure. The central communication to the population from the Civil Protection took several hours to be decided (19). Government high-level meetings were taking place early on, but communication to the population was sparce and there are doubts about the efficiency and timings of the decisions made at a central level (21) Even some days after the blackout, high level National Civil Protection agents in Portugal were still affirming there was no clarity about the problems faced by telecommunication services (19). As a specific example, an SMS message that was sent by the Civil Protection, did not even reach 50% of the available list of contacts, when the usual result is around 95% (19).

### 2.7 Health infrastructure and logistics

Critical medical devices such as ventilators, dialysis machines, CT scanners, and infusion pumps depend on a stable power supply. During the blackout, limited generator capacity forced hospitals to prioritize equipment use. In some cases, devices with internal batteries may have been compromised sooner than expected due to poor maintenance and reduced battery life, an issue that could have been avoided with regular testing and replacement schedules (22). Furthermore, generator capacity can be a problem for the use of such devices, including the days after the blackout, where energy supply is still not normal, imposing a necessity for a triage of equipment usage scenario.

The blackout also disrupted water supply in several hospitals due to electric pump failure, making it impossible to perform haemodialysis in affected units. Without access to purified water, treatments were delayed or cancelled, putting patients at risk. The problem was most acute in older buildings and those at higher elevations, exposing a critical gap in infrastructure readiness. In the same line of thought, greater availability of vehicles to provide drinkable water was felt, since usually these kinds of vehicles are used for other purposes.

Several primary care units and smaller facilities, lacking generators, experienced cold chain failures during the blackout. Temperature-sensitive medicines such as vaccines and insulin were at risk of being lost. In some areas, supplies had to be urgently moved to supermarket refrigerators, as seen in the North of the country, where vaccines and drugs were taken to a Supermarket store due to lack of alternatives (19). Across the country, hospitals also reduced scheduled activities to save energy, and some health centres removed vaccines to avoid spoilage (23). Several emergency transfers to hospital pharmacies were conducted, which was easier due to the recent reform in Portugal that included a vertical integration of primary healthcare facilities and Hospital care under the same administration (24). However, coordination and standard protocols were still undefined and untested in the current organization landscape, leading to potential waste and treatment delays in the following days given the due processes for restocking.

### 2.8 Health and related services

As already pointed out previously, there was a limitation of access to several aspects of the normal provision of care, even regarding emergency services. Due to multiple reasons, at a given point even related to the water supply, several of the healthcare facilities limited their activity. Starting with primary healthcare facilities, there is an overall lack of energy backup. As such, services were limited to the essential procedures (4). To conserve energy, most hospitals suspended elective surgeries, outpatient consultations, diagnostic tests, and non-urgent follow-ups (25). While necessary, these suspensions disrupted care pathways.

However, there were some institutions that were able to continue normal functioning during the time of the blackout (23). In some cases this also lead to a greater pressure in institutions that kept providing specific activities of care.

### 2.9 Community capacities for health EDRM

As already alluded to before, while the constraints about water supplies were also felt in some hospitals, it was mainly in the community setting, that this limitation was more acute. This is due to the fact that it is dependent on the stablished global infrastructure. Primary care units are served by the regular civil services, and the same happens for other vital structures, as for example dialysis clinics. As such, if given areas in the territory are served by water services that are dependent on electric pumps, they were affected even longer than the duration of the blackout as it was also needed to restart the due processes. In some cases, the knowledge about these intrinsic limitations were not known.

Patients receiving hospital-at-home care, particularly those using oxygen concentrators or ventilators, may have been impacted by the blackout, especially since several hours there was no perspective on when the power source would be established again. It is estimated that more than 11.000 patients receive home-care treatment in 2024 (26). Due to this concern, some of these patients ended up in hospitals, adding further pressure on already strained emergency departments and inpatient units. The situation exposed a blind spot in home care resilience planning, at least from a communication standpoint, since in Spain, three people are suspected to have died from carbon monoxide poisoning due to the use of a generator to power an oxygen machine (7).

### 2.10 Monitoring and evaluation

The blackout exposed critical vulnerabilities in the energy autonomy of hospital infrastructure. While diesel generators are the standard in most public hospitals, Portugal lacks a national system for real-time monitoring of their operational status, capacity, and remaining fuel autonomy. This absence of centralized oversight significantly delayed coordinated support efforts. According to the National Authority for Emergency and Civil Protection, it took nearly five hours to establish a complete overview of generator fuel levels and autonomy across the network (19). At Maternidade Alfredo da Costa (Local Health Unit of São José), one of the largest maternity hospitals in the country, the situation escalated to a critical stage when there was only one hour of generator power left, according to the Minister of Territorial Cohesion (25), prompting contingency discussions about repurposing diesel from government vehicles, a measure ultimately avoided but indicative of serious planning deficits and the absence of predefined emergency fuel delivery protocols (27). Furthermore, the Prime-Minister of Portugal admitted that the most difficult to manage in the blackout was the energy supply to hospitals, assuring there were no limit situations, though (27).

## 3 Discussion

The April 2025 Iberian power outage unveiled critical systemic weaknesses embedded in Portugal’s health infrastructure, exposing its fragility as a sovereign and strategic institution. From fuel shortages at maternity hospitals to the failures of digital systems and emergency communication lines, the blackout exposed an unprepared healthcare system for long lasting disruption. These failures do not seem to be isolated or technical oversights, they are symptoms that point to a deeper governance and strategic planning deficit that might threaten national sovereignty.

These infrastructural fragilities compounded one another. Communications breakdown rendered coordination with civil protection entities nearly impossible. The inaccessibility of electronic health records increased clinical risk. Emergency dispatch systems and road-based transport logistics were hampered simultaneously by digital and traffic control failures. The dependence of life-sustaining devices on uninterrupted electricity forced hospitals into triage scenarios. This interdependence of vulnerabilities demonstrates that the health system’s distress was systemic, not circumstantial.

Despite governmental reassurances, operational records and professional accounts tell a different story, as response protocols were triggered late, vulnerabilities were managed *ad hoc*, and coordination was hindered by infrastructure that had not been meaningfully stress-tested prior to the blackout. Indeed, the national emergency response plan was only formally activated after power had already begun to return in several areas, reflecting not only governance delay but a worrying absence of anticipatory planning. The government’s decision to open an EU-level inquiry into the origins of the blackout was appropriate, yet this retrospective stance does little to compensate for the absence of real-time resilience (21).

There is also room for reflection about the feasibility of the leaders’ intent during the recovery of the blackout. While there was a clear will from the government to restore energy supply to Hospitals, and other vital structures, it was evident that this was not followed through. Several areas recovered energy supply before the areas that had critical infrastructures. As such, it would also be important to consider the changes at the level of infrastructure that are needed to comply with the most obvious leadership intentions during these scenarios. Likewise, greater reflections are needed in urban planning to assure that in moments of greater congestion and gridlocks, there are routs that should be used preferably by and for essential services. The current moment is also particularly relevant given the high level of investment that is being done with the Portugal’s Recovery and Resilience Plan. Assurance that the new buildings that are being built, for example, have the proper infrastructure capability is key. And future funds should also address the need to renew old infrastructures, where needed.

The blackout should be reflected upon as a diagnostic moment, particularly considering the recent launch of the European Commission’s Preparedness Union Strategy, where some of the points raised in this manuscript find resonance. In a geopolitical climate where energy security, digital sovereignty, and public health resilience increasingly define national preparedness, Portugal must reconceptualize its health infrastructure as an essential domain of strategic autonomy. The evidence gathered from this event reveals the consequences of lack of planning, underinvestment in readiness, and absence of integrated policy linking health and security.

Central to this transformation is the question of energy autonomy. The dependence on diesel generators, many of which were unmonitored or nearing depletion, proved unsustainable. The integration of decentralized, renewable-energy-based systems, such as solar arrays with battery storage, offers a clear path toward sustained operational capacity during grid failure. These systems, as recognized by the World Health Organization, enhance institutional autonomy, reduce reliance on vulnerable fuel supply chains, and provide continuous support to critical functions (28). Solar-plus-battery systems enhance hospital resilience by ensuring power continuity during outages, unlike diesel generators, which are vulnerable to fuel shortages. WHO highlights such renewable setups as essential for uninterrupted healthcare delivery and energy autonomy (28).

To build a comprehensive, cross-sectoral response, Portugal should institutionalize an integrated command structure linking the Ministries of Health, Internal Administration, and Defence. This structure must conduct crisis simulation exercises, manage national medical reserves, and ensure interagency interoperability, elements already considered best practices in NATO and EU resilience doctrines (29, 30). Simultaneously, the NHS should adopt robust digital redundancy protocols, including offline EHR access, network failovers, and decentralized data security.

The April 2025 blackout is a sentinel event. Even though it triggered fears of potential impacts of new, convergent risks such as cyberattacks, climate-induced disasters, and hybrid threats, this is not a new risk (31). While Ukraine’s recent expertise with restoring power grids was even offered to Portugal and Spain (32), there are several lessons from previous events that should be considered in the aftermath of the blackout and needed preparedness, such as the blackout in North America of August 2003, among others (33, 34). It is vital that a formal review of what happened takes place. However, such work will only benefit from rapid reviews such as this one, even if the sources provided are mostly based in grey literature. Without a fundamental reorientation of planning doctrines, regulatory instruments, and investment strategies, the NHS will remain exposed to shocks that undermine its functionality and legitimacy.

Health sovereignty, in this sense, must be entrenched not as an aspirational value, but as a condition of national survival. Failure to do so will leave Portugal vulnerable to blackouts and any threat capable of destabilizing the fragile equilibrium on which its healthcare and national continuity depend.

## 4 Conclusion

The April 2025 Iberian power outage served as a definitive stress test for Portugal’s NHS, exposing a convergence of structural, digital, and operational vulnerabilities that critically undermined healthcare delivery during crisis. Far from being a discrete infrastructure failure, the blackout seems to have revealed the cumulative effects of fragmented contingency planning, outdated emergency logistics, and deficient inter-ministerial coordination. A proper formal evaluation should take place and this perspective article aims to contribute to the necessary discussion with the first-hand experience of several experts in the field, as well as the grey literature available.

The resilience of health systems must be reframed not as a secondary administrative concern but as a strategic imperative embedded in national security frameworks. Emergency fuel logistics, digital health infrastructure redundancy, telecommunications resilience, and cold storage infrastructure represent interconnected domains that cannot be coordinated in isolation. The reactive nature of the Portuguese response, characterized by delayed decision-making, asymmetric resource allocation, and an absence of central coordination, illustrates the inadequacy of existing governance frameworks to support large-scale emergency response.

Given the geopolitical environment and the European Commission’s recent launch of the Preparedness Union Strategy, this event provides a critical inflection point. Portugal must institutionalize health sovereignty as a principle of governance, anchored in energy autonomy, robust data infrastructure, interoperable logistics chains, and multisectoral crisis governance. This requires immediate legislative action to enforce service continuity standards, substantial investment in strategic infrastructure, and the integration of defence, civil protection, and health authorities in shared simulation and preparedness protocols.

If health systems are to fulfil their dual mandate, clinical care and societal protection, they must be operationally self-reliant and strategically embedded in national security frameworks. The Iberian blackout’s lessons must now catalyze a transformation: from a segmented, reactive healthcare service to an anticipatory system capable of withstanding systemic shocks without compromising its sovereignty or the safety of its population. Only by translating diagnostic reflection into structural reform will Portugal be equipped to navigate an era defined by complex and emerging crises.

## Data Availability

The original contributions presented in the study are included in the article/supplementary material, further inquiries can be directed to the corresponding author/s.
